# Eye-Tracking Algorithm for Early Glaucoma Detection: Analysis of Saccadic Eye Movements in Primary Open-Angle Glaucoma

**DOI:** 10.3390/jemr18030018

**Published:** 2025-05-19

**Authors:** Cansu Yuksel Elgin

**Affiliations:** Cerrahpasa Faculty of Medicine, Department of Ophthalmology, Istanbul University, Cerrahpaşa, Koca Mustafapaşa Cd. No:53, Fatih, 34098 İstanbul, Turkey; cansu.elgin@iuc.edu.tr; Tel.: +90-537-5033505

**Keywords:** glaucoma, primary open-angle glaucoma, eye tracking, saccadic eye movements, motion perception, oculomotor behavior, early detection, machine learning algorithm, preperimetric glaucoma, visual function

## Abstract

Glaucoma remains a leading cause of irreversible blindness worldwide, with early detection crucial for preventing vision loss. This study developed and validated a novel eye-tracking algorithm to detect oculomotor abnormalities in primary open-angle glaucoma (POAG). We conducted a case–control study (March–June 2021), recruiting 16 patients with moderate POAG, 16 with preperimetric POAG, and 16 age-matched controls. The participants underwent a comprehensive ophthalmic examination and eye movement recording using a high-resolution infrared tracker during two tasks: saccades to static targets and saccades to moving targets. The patients with POAG exhibited a significantly increased saccadic latency and reduced accuracy compared to the controls, with more pronounced differences in the moving target task. Notably, preperimetric POAG patients showed significant abnormalities despite having normal visual fields based on standard perimetry. Our machine learning algorithm incorporating multiple saccadic parameters achieved an excellent discriminative ability between glaucomatous and healthy subjects (AUC = 0.92), with particularly strong performance for moderate POAG (AUC = 0.97) and good performance for preperimetric POAG (AUC = 0.87). These findings suggest that eye movement analysis may serve as a sensitive biomarker for early glaucomatous damage, potentially enabling earlier intervention and improved visual outcomes.

## 1. Introduction

Glaucoma represents a significant global health concern, ranking as a leading cause of irreversible blindness worldwide [1]. Characterized by progressive optic neuropathy and distinctive visual field defects, primary open-angle glaucoma (POAG) in particular constitutes the most prevalent form of the disease, often remaining asymptomatic until substantial damage has occurred [2]. Current diagnostic methods predominantly rely on detecting structural changes to the optic nerve head and measuring the visual field loss through perimetry, approaches that frequently identify the disease only after significant neuronal damage has already occurred [3].

The detection of subtle functional changes in early glaucoma remains challenging, creating a critical need for innovative diagnostic approaches that can identify the disease before irreversible damage ensues. In recent years, the analysis of oculomotor behavior has emerged as a promising avenue for early glaucoma detection, with several studies demonstrating altered eye movement patterns in patients with established glaucomatous damage [4,5]. These findings suggest that eye movement analysis could potentially serve as a sensitive biomarker for detecting early neuronal dysfunction in glaucoma.

The relationship between glaucoma and oculomotor function is rooted in the disease’s pathophysiology. Glaucoma primarily damages retinal ganglion cells (RGCs) and their axons. Although earlier studies, such as that by Quigley et al. (1987) [6], focused predominantly on retinal nerve fiber loss, the more recent literature has indeed identified differential vulnerability among RGC subpopulations (e.g., parasol cells linked to the magnocellular pathway). Recent human studies have supported the presence of selective damage to these RGC subsets early in glaucoma development, underscoring the relevance of targeting specific visual pathways in diagnostic assessments [7]. Of particular interest is the potential vulnerability of the magnocellular pathway, which plays a crucial role in motion detection and spatial localization [8]. Several studies have documented impaired motion perception in glaucoma patients, implicating dysfunction within this visual subsystem [9,10]. Since accurate eye movements, particularly saccades, rely heavily on intact motion processing mechanisms, this pathway being compromised may manifest as detectable oculomotor anomalies.

Saccades—rapid, conjugate eye movements that redirect the fovea toward visual targets—represent a particularly promising parameter for investigation in glaucoma. These movements require precise neural coordination across multiple brain regions, including the superior colliculus, which receives direct input from RGCs and serves as a critical hub for saccade programming [11]. The integrity of this pathway may thus reflect the health of the visual system more broadly. It has also been demonstrated that glaucoma patients exhibit altered saccadic parameters, including an increased latency and decreased accuracy, particularly when tracking moving targets [5]. Importantly, these abnormalities were detectable even in preperimetric glaucoma patients who had not yet developed visual field defects detectable using standard clinical testing.

Building upon these promising findings, we propose that advanced computational algorithms applied to eye-tracking data could potentially detect subtle oculomotor changes associated with early glaucomatous damage. Machine learning approaches have shown considerable success in detecting patterns within complex biological data that may not be immediately apparent when using conventional statistical methods [12]. By identifying distinctive patterns in eye movement behavior, such algorithms might capture early functional changes before standard clinical tests reveal abnormalities. Our research addressed a significant gap in current glaucoma diagnostics by developing and validating a novel algorithm specifically designed to detect characteristic oculomotor anomalies in glaucoma. This approach offers several potential advantages: it is non-invasive, provides objective measurements, and can potentially detect functional changes that precede structural damage. The ability to identify glaucomatous changes at an earlier stage would represent a significant advancement in clinical care, potentially allowing for earlier intervention and improved visual outcomes.

To summarize, in this study, we present the development and validation of an innovative eye-tracking algorithm designed to detect glaucomatous changes through the analysis of saccadic eye movements. By examining both the static and dynamic task performance across patient cohorts with varying disease severity, we aimed to establish the sensitivity and specificity of this approach for early glaucoma detection.

## 2. Materials and Methods

This observational case–control study was designed to investigate the diagnostic potential of oculomotor abnormalities in glaucomatous patients. The study protocol was approved by the Institutional Review Board of Sisli Hamidiye Etfal Training and Research Hospital (approval number: 2020-356) and conducted in accordance with the Declaration of Helsinki. Written informed consent was obtained from all participants prior to enrollment. Data collection was performed between March and June 2021 in the Department of Ophthalmology.

A total of 48 individuals were recruited for this study, comprising three distinct groups: 16 patients with moderate primary open-angle glaucoma (POAG), 16 patients with preperimetric POAG, and 16 age-matched healthy controls. The participants were between 40 and 70 years of age. The inclusion criteria for all participants were (1) a best-corrected visual acuity of ≥20/25 in the study eye, (2) a refractive error within a ±6.00 diopters spherical equivalent and ±2.00 diopters of astigmatism, (3) no history of ocular or neurological conditions other than glaucoma in the glaucoma groups, and (4) no history of ocular surgery except for uncomplicated cataract surgery performed at least 6 months prior to enrollment. Participants in the moderate POAG group had (1) glaucomatous optic neuropathy with characteristic optic disk changes and retinal nerve fiber layer (RNFL) thinning as documented by optical coherence tomography (OCT), (2) reproducible visual field defects consistent with glaucoma (defined as a cluster of ≥3 points with *p* < 0.05 on a pattern deviation map, with at least one point with *p* < 0.01), and (3) open angles according to gonioscopy. The mean deviation in Humphrey Visual Field testing ranged from −6.0 to −12.0 dB. Participants in the preperimetric POAG group had (1) glaucomatous optic neuropathy with characteristic optic disk changes and RNFL thinning according to OCT, (2) an intraocular pressure of >21 mmHg prior to treatment, (3) normal visual fields according to standard automated perimetry (defined as a mean deviation > −2.0 dB with no significant points in the pattern deviation probability plot), and (4) open angles according to gonioscopy. The control subjects had (1) normal findings based on an ophthalmic examination, (2) an intraocular pressure of ≤21 mmHg, (3) normal-appearing optic disks with a cup-to-disk ratio of ≤0.4, (4) a normal RNFL thickness according to OCT, and (5) normal visual fields. The exclusion criteria for all groups included (1) a history of neurological or psychiatric disorders, (2) the use of medications known to affect eye movements (e.g., benzodiazepines, neuroleptics, anticonvulsants), (3) alcohol consumption within 24 h prior to testing, (4) an inability to maintain stable fixation, and (5) a poor comprehension of the test instructions.

All participants underwent a comprehensive ophthalmic examination including the measurement of their best-corrected visual acuity, slit lamp biomicroscopy, Goldmann applanation tonometry, gonioscopy, a dilated fundus examination, and standard automated perimetry (Humphrey Field Analyzer II 750; Carl Zeiss Meditec, Dublin, CA, USA) using the 24-2 SITA Standard program. Spectral-domain OCT (Cirrus HD-OCT; Carl Zeiss Meditec, Dublin, CA, USA) was performed to measure the peripapillary RNFL thickness and macular ganglion cell complex parameters. Imaging was performed by a trained technician masked to the participants’ clinical status. Only high-quality scans with a signal strength of ≥7 and without movement artifacts or segmentation errors were included in the analysis.

Eye movements were recorded using a desktop-mounted infrared video-based eye tracker (EyeLink 1000 Plus; SR Research Ltd., Ottawa, ON, Canada) with a sampling rate of 1000 Hz and a spatial resolution of <0.01°. Participants were seated 65 cm from a 24-inch LCD monitor (1920 × 1080 pixel resolution, 144 Hz refresh rate) in a quiet, dimly lit room. Head movements were minimized using a chin and forehead rest. Testing was conducted monocularly in the right eye for all participants, with the fellow eye occluded with an opaque patch. The right eye was selected for testing consistency, as previous research has shown that ocular dominance and eye-tracking measurements generally do not differ significantly between the eyes. This standardization reduced the variability and simplified the experimental protocol. A 9-point calibration and validation procedure was performed at the beginning of each session and repeated if the average error exceeded 0.5°. Drift correction was performed before each trial to compensate for potential shifts in the head position.

We selected a Random Forest classifier due to its robustness in handling relatively small datasets, resistance to overfitting, and interpretability through feature importance metrics. Additionally, Random Forest models can manage correlated predictors effectively, which was advantageous given our multiple related saccadic features. Feature selection was conducted in a data-driven manner using the permutation importance to identify features that contributed significantly to the classification performance. The hyperparameters for the Random Forest model were optimized using a grid search approach (number of trees: 100, 200, 500; maximum depth: 3–10; minimum samples per split: 2–10). Optimal parameters were chosen based on the maximum cross-validation accuracy within the nested cross-validation framework. Nested cross-validation (inner loop: hyperparameter tuning; outer loop: performance estimation) was utilized to provide unbiased performance estimates and avoid overfitting. Class balancing was ensured by having equal numbers of subjects in each group (16 participants per class: moderate POAG, preperimetric POAG, and controls), eliminating potential bias in the classification performance. Training and testing sets were strictly separated by the participants, ensuring that data from a single individual did not simultaneously appear in the training and validation sets, thus rigorously preventing data leakage.

Visual stimuli were generated using MATLAB (’MATLAB R2023a, MathWorks, Natick, MA, USA) with the Psychophysics Toolbox extensions. Participants performed two distinct oculomotor tasks. The first one was the saccade to static targets task. This task assessed basic saccadic parameters during a saccade to stationary targets. Each trial began with a central fixation cross (0.5° × 0.5°) being presented for a random duration of between 1000 and 1500 ms. Then, a peripheral target (a white dot, 0.5° in diameter) appeared at one of eight possible locations (at 0°, 45°, 90°, 135°, 180°, 225°, 270°, or 315°) with an eccentricity of 10° from the central fixation. The target remained visible for 1000 ms. Participants were instructed to look at the target as quickly and accurately as possible. A total of 64 trials were performed (8 trials for each target location) in a pseudo-randomized order.

The second task was the saccade to moving targets. This task evaluated participants’ ability to execute saccades toward moving targets. Each trial began with a central fixation cross being displayed for 1000–1500 ms. A peripheral target then appeared at an eccentricity of 15° and immediately began moving toward the center at one of three possible velocities (5°/s, 10°/s, or 15°/s). The central fixation cross disappeared 200 ms after the peripheral target began moving, signaling participants to make a saccade to the moving target and track it. The target continued moving through the center point and to the opposite side of the screen. A total of 72 trials were performed (8 trials for each of the 3 velocities in each of the 3 directions) in a randomized order.

**Figure 1:**
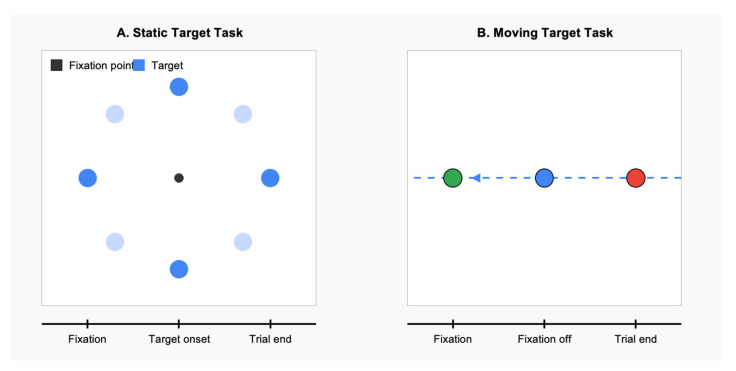
Experimental paradigm for eye movements tasks (**A**) Static Target Task and (**B**) Moving Target Task.

Figure 1 illustrates the two eye movement tasks employed in this study. Panel A depicts the static target task. Panel B shows the moving target task. These complementary paradigms allowed for the assessment of saccadic parameters under both static and dynamic visual conditions.

For data processing, the obtained eye movement data were processed offline using custom algorithms developed in Python 3.8 using the NumPy, SciPy, and scikit-learn libraries. Saccades were detected using a velocity-based algorithm with a threshold of 30°/s. The onset of a saccade was defined as the first point where the velocity exceeded this threshold, and the offset was defined as the point where the velocity returned below this threshold. For each saccade, we extracted the following parameters: the latency, amplitude, peak velocity, duration, and endpoint accuracy (defined as the Euclidean distance between the saccade endpoint and the target position). For the moving target task, we also calculated the intercept accuracy (defined as the distance between the saccade endpoint and the position of the moving target at the time of saccade completion). Based on these parameters, we developed a machine learning algorithm to distinguish between glaucomatous and healthy subjects. We employed a Random Forest classifier with 10-fold cross-validation to evaluate its performance. The feature importance was assessed using permutation importance metrics.

Finally, statistical analyses were performed using R version 4.0.3 (R Foundation for Statistical Computing, Vienna, Austria). Demographic and clinical characteristics were compared across groups using a one-way ANOVA for continuous variables and chi-square tests for categorical variables. Post hoc comparisons were performed using Tukey’s HSD test. For oculomotor parameters, we employed linear mixed-effects models with the participants as random effects and the group, target location/direction, and velocity (for moving targets) as fixed effects. Age was included as a covariate in all models. Receiver operating characteristic (ROC) curve analysis was used to assess the diagnostic performance of individual parameters and the combined algorithm. Statistical significance was set at *p* < 0.05, with Bonferroni correction for multiple comparisons when appropriate.

## 3. Results

In terms of the demographics, a total of 48 participants successfully completed the study protocol and were included in the final analysis. The demographic and clinical characteristics of the three groups are summarized in Table 1. There were no significant differences in the age (*p* = 0.68), gender distribution (*p* = 0.83), or best-corrected visual acuity (*p* = 0.47) between groups. As expected, significant differences were observed in the mean deviation in the results from visual field testing between the moderate POAG group (−8.56 ± 2.31 dB) and both the preperimetric POAG group (−0.87 ± 0.93 dB, *p* < 0.001) and control group (−0.23 ± 0.79 dB, *p* < 0.001). Similarly, the average RNFL thickness was significantly reduced in the moderate POAG group (68.3 ± 9.1 μm) compared to the preperimetric POAG group (82.7 ± 7.3 μm, *p* < 0.001) and control group (96.2 ± 6.8 μm, *p* < 0.001). The preperimetric POAG group also showed significant RNFL thinning compared to the controls (*p* < 0.001), despite having normal visual fields.

Next, the analysis of the saccade to static targets task revealed significant differences in the saccadic parameters between groups. The moderate POAG group demonstrated an increased saccadic latency (256 ± 31 ms) compared to both the preperimetric POAG group (237 ± 28 ms, *p* = 0.042) and control group (221 ± 24 ms, *p* < 0.001). Interestingly, the preperimetric POAG group also showed significantly longer latencies than the control group (*p* = 0.038), suggesting that delayed saccadic initiation may be detectable even in early-stage disease. The saccadic gain (the ratio of the saccade amplitude to the target eccentricity) was significantly reduced in the moderate POAG group (0.83 ± 0.09) compared to both the preperimetric POAG group (0.91 ± 0.07, *p* = 0.008) and control group (0.94 ± 0.06, *p* < 0.001), indicating hypometric saccades in patients with more advanced disease. The preperimetric POAG group did not differ significantly from controls in terms of the saccadic gain (*p* = 0.26). The peak saccadic velocity was significantly lower in the moderate POAG group (336 ± 41°/s) compared to the controls (371 ± 38°/s, *p* = 0.006), but no significant difference was observed between the preperimetric POAG group (352 ± 43°/s) and the controls (*p* = 0.18). The saccadic duration did not differ significantly between groups (*p* = 0.39). The endpoint accuracy, measured as the distance between the saccade endpoint and the target position, was significantly worse in the moderate POAG group (1.87 ± 0.68°) compared to controls (1.14 ± 0.43°, *p* < 0.001). The preperimetric POAG group showed intermediate values (1.42 ± 0.52°), which differed significantly from both the moderate POAG group (*p* = 0.032) and the control group (*p* = 0.047).

The second task, the saccade to moving targets, revealed more pronounced differences between the groups than the static target task. The saccadic latency in response to moving targets was markedly increased in the moderate POAG group (312 ± 43 ms) compared to both the preperimetric POAG group (271 ± 35 ms, *p* < 0.001) and the control group (234 ± 29 ms, *p* < 0.001). The preperimetric POAG group also showed significantly delayed saccades compared to the controls (*p* = 0.003). The intercept accuracy, which measures the ability to accurately intercept a moving target, was significantly impaired in both glaucoma groups. The moderate POAG group showed the poorest performance (3.64 ± 1.12°), followed by the preperimetric POAG group (2.78 ± 0.93°) and control group (1.95 ± 0.67°), with significant differences between all pairs of groups (all *p* < 0.01). The target velocity had a significant effect on the saccadic parameters across all groups, with higher velocities associated with a worse intercept accuracy and shorter saccadic latencies. Notably, the differences between groups became more pronounced at higher target velocities (10°/s and 15°/s) compared to the lowest velocity (5°/s), suggesting that more challenging oculomotor tasks may better discriminate between glaucomatous and healthy individuals.

**Figure 2:**
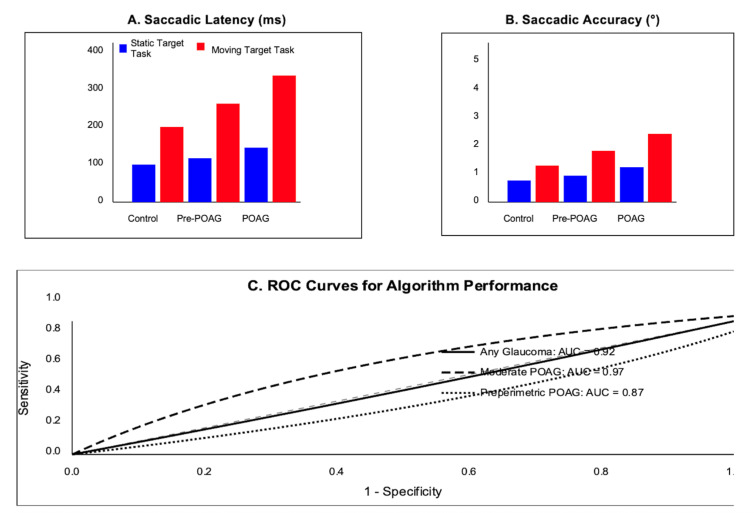
Comparison of saccadic parameters, (**A**) Saccadic Latency (ms), (**B**) Saccadic Accuracy, (**C**) ROC Curves for Algorithm Performance.

Figure 2 presents the key saccadic parameters across the three participant groups. Panel A shows the saccadic latency measurements for both the static and moving target tasks. The latencies became progressively longer from the control subjects (static: 221 ± 24 ms; moving: 234 ± 29 ms) to the preperimetric POAG patients (static: 237 ± 28 ms; moving: 271 ± 35 ms) and moderate POAG patients (static: 256 ± 31 ms; moving: 312 ± 43 ms), with the differences being more pronounced in the moving target task. Panel B illustrates the saccadic accuracy measured as the endpoint error in degrees. The accuracy progressively worsened from the controls to the moderate POAG patients, with larger errors observed in the moving target task. Panel C displays the receiver operating characteristic (ROC) curves for the algorithm’s diagnostic performance, showing excellent discrimination for moderate POAG (AUC = 0.97), good discrimination for any glaucoma (AUC = 0.92), and promising results for detecting preperimetric POAG (AUC = 0.87). The statistical analysis revealed significant differences between all the groups (*p* < 0.05), with the most substantial differences observed between the moderate POAG and control groups (*p* < 0.001).

**Figure 3:**
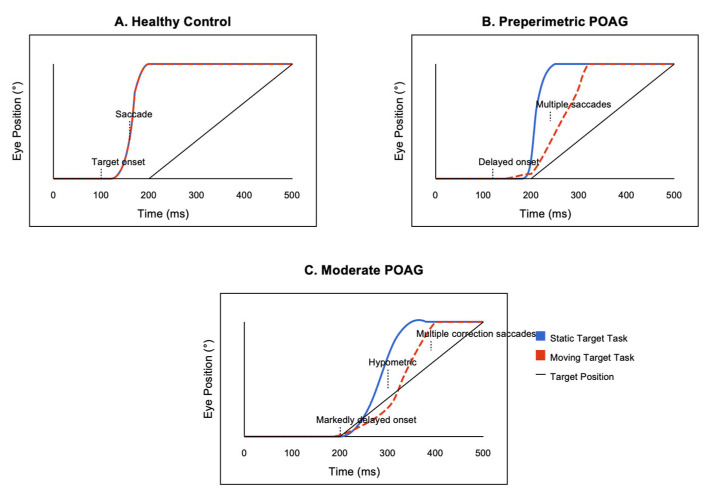
Representative eye movement traces, (**A**) Healthy Control, (**B**) Preperimetric POAG, (**C**) Moderate POAG.

Figure 3 shows representative eye movement recordings from the three participant groups. Panel A displays traces from a healthy control subject, demonstrating prompt saccade initiation (latency of ~220 ms) and accurate target acquisition with a single saccade for both static and moving targets. Panel B illustrates eye movements from a preperimetric POAG patient, showing delayed saccade initiation (latency of ~240 ms for static and ~270 ms for moving targets) and occasional corrective saccades, particularly for moving targets. Panel C depicts traces from a moderate POAG patient, revealing markedly delayed saccade initiation (latency of >250 ms for static and >300 ms for moving targets), hypometric initial saccades that fell short of the target, and multiple corrective saccades needed to eventually reach the target position. These characteristic patterns were consistent across the participants within each group and highlight the progressive deterioration of oculomotor control with increasing glaucoma severity. While Figure 3 visually demonstrates similar final target acquisition times across the groups, it is important to clarify that the moderate POAG group typically required multiple corrective saccades to reach the target. Although the final endpoint timing appears similar, the latency of the initial saccade onset was significantly delayed, indicating impaired initial saccade accuracy and efficiency.

Regarding the classification performance of the algorithm, the machine learning algorithm incorporating multiple saccadic parameters demonstrated an excellent ability to discriminate between glaucomatous and control subjects. The area under the ROC curve (AUC) for distinguishing participants with any glaucoma (combining the moderate and preperimetric POAG groups) from the controls was 0.92 (95% CI: 0.84–0.97). When analyzed according to the disease severity, the algorithm showed outstanding performance in distinguishing patients with moderate POAG from the controls (AUC = 0.97; 95% CI: 0.91–0.99) and good performance in identifying preperimetric POAG (AUC = 0.87; 95% CI: 0.77–0.94). Using the optimal threshold derived from the ROC analysis, the algorithm achieved a sensitivity of 88.5% and specificity of 86.7% for detecting any glaucoma. For moderate POAG, the sensitivity and specificity were 93.8% and 93.8%, respectively, while for preperimetric POAG, these values were 81.3% and 87.5%. Feature importance analysis revealed that the saccadic parameters derived from the moving target task, particularly the intercept accuracy and latency at higher target velocities, contributed most significantly to the algorithm’s performance. This finding underscores the value of complex oculomotor tasks in enhancing diagnostic sensitivity. Correlation analysis showed significant relationships between certain saccadic parameters and conventional glaucoma metrics. Specifically, the intercept accuracy in the moving target task correlated with both the mean deviation in the results from visual field testing (r = −0.68, *p* < 0.001) and the average RNFL thickness (r = −0.71, *p* < 0.001), suggesting that oculomotor performance may reflect the extent of glaucomatous damage. Preliminary analyses comparing Random Forest with other classifiers, such as support vector machines (SVMs) and logistic regression, indicated that Random Forest provided the most stable and highest accuracy (RF: AUC = 0.92; SVM: AUC = 0.85; logistic regression: AUC = 0.81), supporting our final choice of classifier. The performance metrics were consistent across the cross-validation folds, with the standard deviation of the AUC across folds remaining below 0.05, indicating stable and reliable model performance.

## 4. Discussion

Our study demonstrates that patients with primary open-angle glaucoma (POAG) exhibit significant abnormalities in their saccadic eye movements, particularly when executing saccades toward moving targets. The novel eye-tracking algorithm we developed successfully distinguished between glaucomatous and healthy subjects with high sensitivity and specificity, suggesting potential clinical utility as a diagnostic tool for the early detection of glaucoma. Importantly, our findings indicate that oculomotor abnormalities can even be detected in patients with preperimetric glaucoma who have not yet developed visual field defects according to standard automated perimetry, highlighting the potential of eye movement analysis as a sensitive biomarker for early glaucomatous damage.

The observed increase in the saccadic latency among glaucoma patients aligns with previous research findings. Delayed saccades have been reported in POAG patients, with more pronounced delays when tracking moving targets compared to static ones [5]. Our results extend these observations by demonstrating a gradient of impairment that correlates with the disease severity, with preperimetric patients showing intermediate values between those of controls and those of patients with moderate glaucoma. This pattern suggests that saccadic delay may progressively worsen as the disease advances, potentially reflecting the cumulative effect of glaucomatous damage on neural pathways critical for oculomotor control.

The mechanisms underlying these oculomotor abnormalities likely involve the disruption of specific visual processing streams affected by glaucoma. The magnocellular pathway, implicated in motion processing and spatial localization, has been proposed to be preferentially vulnerable in early glaucoma. Although previous studies, such as that by McKendrick et al. (2007) [13], primarily addressed contrast sensitivity disruptions, the additional literature indicates that magnocellular dysfunction could specifically disrupt visual motion perception and oculomotor responses [8,9,10]. Thus, the abnormalities observed in saccadic behavior may reflect broader dysfunction within magnocellular-driven visual processing networks. This pathway provides essential inputs to cortical and subcortical structures involved in saccade programming, including the superior colliculus, which receives direct projections from retinal ganglion cells [11]. Notably, the superior colliculus is essential for the transformation of visual signals into oculomotor commands and plays a critical role in target selection for saccades [14]. Dysfunction in this pathway could explain the observed delays in saccade initiation and reduced accuracy, particularly for moving targets that impose greater demands on motion processing.

The more pronounced deficits observed during the moving target task compared to the static target task deserve particular attention. The execution of saccades toward moving targets requires additional neural computations, including accurate motion analysis and the prediction of the target’s future position [15]. These processes rely heavily on intact motion processing mechanisms that may be compromised in glaucoma. A previous study reviewed numerous studies demonstrating impaired motion perception in glaucoma patients, even in the absence of significant visual field defects [8]. Our findings are consistent with this literature and suggest that tasks requiring motion analysis may be particularly sensitive for detecting early functional changes in glaucoma.

The reduced saccadic gain and endpoint accuracy observed in glaucoma patients may reflect impaired spatial localization abilities. Previous research [16] has shown that patients with glaucoma demonstrate difficulties in localizing peripheral targets, which could have contributed to the hypometric saccades observed in our study. The neural basis for these spatial deficits likely involves both retinal and post-retinal mechanisms. At the retinal level, the loss of ganglion cells results in a reduced sampling density and potential distortions in the spatial representation of the visual field. At the post-retinal level, structural changes have been documented [17] in the lateral geniculate nucleus and visual cortex of glaucoma patients, which could further compromise spatial processing.

Interestingly, our feature importance analysis revealed that the parameters derived from the moving target task contributed most significantly to the algorithm’s diagnostic performance. This finding aligns with the growing recognition that dynamic visual tasks may be more sensitive than static ones for detecting functional vision loss in glaucoma. A study demonstrated that motion discrimination thresholds were elevated in glaucoma patients, even in regions of the visual field classified as normal according to standard perimetry [18]. Similarly, another study found that performance on a motion-defined form task was significantly impaired in glaucoma and correlated with self-reported difficulties in daily activities [19]. Our results extend these observations to the domain of oculomotor control, suggesting that eye movement responses to moving stimuli may provide a sensitive window into early visual dysfunction in glaucoma.

The strong correlation between the saccadic parameters and conventional glaucoma metrics (mean deviation and RNFL thickness) suggests that oculomotor performance reflects the underlying extent of glaucomatous damage. This relationship supports the potential clinical utility of eye movement analysis as an objective measure of functional impairment. Traditional perimetry, while valuable, has known limitations, including subjectivity, patient fatigue, and learning effects [20]. Eye tracking offers several advantages as a complementary diagnostic tool, including objective measurement, the potential for higher sensitivity to early changes, and the ability to probe specific visual functions not adequately assessed by conventional perimetry.

Also, the high diagnostic performance of our algorithm, particularly for preperimetric glaucoma (AUC = 0.87), is promising and compares favorably with other emerging technologies for early glaucoma detection. For context, studies [21] on using optical coherence tomography angiography (OCTA) for detecting preperimetric glaucoma have reported AUC values ranging from 0.73 to 0.89, while electroretinography studies have achieved AUCs of 0.73 to 0.85 [22]. Our approach offers the additional advantage of providing direct insight into the functional visual performance, potentially bridging the gap between structural and functional assessments in glaucoma.

Although the present study demonstrated strong diagnostic accuracy, particularly for preperimetric glaucoma, its clinical integration requires the clear delineation of the target population and the consideration of specificity. Our eye-tracking algorithm is specifically intended for screening glaucoma suspects—individuals presenting with risk factors such as an elevated intraocular pressure, optic disk abnormalities, or suspicious retinal nerve fiber layer thinning. Given the algorithm’s demonstrated sensitivity to early functional impairments, it is envisioned as a non-invasive, initial screening tool to identify individuals who warrant detailed ophthalmologic evaluation. Eye tracking could complement the current diagnostic methods by serving as a rapid, non-invasive, and objective screening tool, particularly useful in initial screenings of glaucoma suspects. While it may not fully replace standard tests (e.g., OCT, perimetry), it can identify subtle functional impairments warranting further evaluation. The practical implementation of our setup in clinical workflows is feasible due to its non-invasive nature, quick test duration (~15 min), and ease of use. Nonetheless, clinician training and protocol standardization would be essential for its broad adoption. We acknowledge, however, that saccadic abnormalities are not exclusive to glaucoma and may arise from numerous neurological and ophthalmologic disorders. Therefore, positive screening outcomes must be interpreted cautiously and combined with the results from established ophthalmologic diagnostic methods to confirm glaucoma. Further research involving a larger and more diverse cohort—including patients with potentially confounding conditions—is essential to establish definitive clinical specificity and reinforce this method’s practical utility in glaucoma management. Future studies are also necessary to externally validate our findings in larger, independent cohorts to confirm generalizability and clinical applicability.

Several limitations of our study warrant discussion. First, our sample size, while adequate for demonstrating significant differences between the groups, was relatively modest. Larger studies will be needed to validate the algorithm’s performance across diverse patient populations. Second, we focused on a limited set of eye movement tasks; future work could explore a broader range of oculomotor behaviors, including smooth pursuit, optokinetic nystagmus, and more complex scanning patterns during naturalistic viewing. Third, while we controlled for age, other factors such as patients’ cognitive status, which can influence saccadic performance, were not comprehensively assessed. Finally, the cross-sectional design limited our ability to determine whether oculomotor abnormalities predict the future development or progression of glaucoma; longitudinal studies will be essential to address this question. Despite these limitations, our findings have important clinical implications. The ability to detect functional visual abnormalities in preperimetric glaucoma could facilitate earlier diagnosis and treatment, potentially preserving vision that might otherwise be lost. Furthermore, the quantitative assessment of oculomotor performance might provide a sensitive measure of disease progression or the response to treatment. From a practical perspective, eye-tracking technology has become increasingly accessible, with numerous commercial systems now available at a relatively modest cost. The integration of oculomotor assessment into clinical practice could therefore be feasible without imposing significant economic or logistical burdens. Finally, our study acknowledges potential confounders such as the cognitive load, fatigue, learning effects, and fixation stability. To mitigate these factors, we implemented short breaks between tasks, randomized the trial orders, and provided carefully formulated participant instructions. However, future studies should explicitly measure these factors and incorporate them as covariates to strengthen the validity of results.

To summarize the main contributions of our study, our study demonstrated that patients with POAG exhibit significant abnormalities in their saccadic eye movements, particularly when executing saccades toward moving targets. These oculomotor deficits could be detected even in patients with preperimetric glaucoma and correlated with conventional metrics of disease severity. Our machine learning algorithm effectively distinguished between glaucomatous and healthy subjects, suggesting potential clinical utility as a diagnostic tool. Future research should focus on longitudinal validation, the refinement of testing protocols, and the exploration of additional eye movement parameters to further enhance diagnostic accuracy. Modern eye-tracking systems are becoming increasingly affordable and user-friendly, potentially making this approach cost-effective for use in larger screening programs. However, detailed economic analyses and workflow integration studies will be required. Eye movement analysis represents a promising approach for early glaucoma detection that may complement the existing structural and functional assessments.

## 5. Conclusions

This study demonstrated that patients with primary open-angle glaucoma exhibit significant abnormalities in their saccadic eye movements, particularly when tracking moving targets. Our novel eye-tracking algorithm successfully distinguished between glaucomatous and healthy subjects with high sensitivity and specificity (88.5% and 86.7%, respectively). Importantly, these oculomotor deficits were detectable even in preperimetric glaucoma patients who had not yet developed visual field defects according to standard automated perimetry. The strong correlation between saccadic parameters and conventional glaucoma metrics suggests that eye movement analysis provides a window into the functional consequences of glaucomatous damage. These findings support the potential clinical utility of eye-tracking technology as a complementary diagnostic tool for early glaucoma detection, which could enable earlier intervention and improved visual outcomes. Future longitudinal studies are warranted to validate these findings in diverse populations and determine whether oculomotor abnormalities can predict disease progression.

## Figures and Tables

**Table 1:** Demographic and clinical characteristics of study participants.

Characteristic	Control (n = 16)	Preperimetric POAG (n = 16)	Moderate POAG (n = 16)	*p*-Value	Effect Size (η^2^)
Age (Years)	55.4 ± 6.2	57.2 ± 5.8	58.9 ± 6.5	0.68	0.02
Gender (Male/Female)	7-Sep	8-Aug	7-Sep	0.83	0.01 *
BCVA (logMAR)	0.03 ± 0.05	0.05 ± 0.06	0.06 ± 0.07	0.47	0.03
IOP (mmHg)	16.2 ± 2.4	17.8 ± 2.1 ^a^	18.5 ± 3.2 ᵃ	0.04	0.13
Mean Deviation (dB)	−0.23 ± 0.79	−0.87 ± 0.93	−8.56 ± 2.31 ᵃᵇ	<0.001	0.84
Pattern Standard Deviation (dB)	1.52 ± 0.48	1.94 ± 0.62	8.72 ± 2.58 ᵃᵇ	<0.001	0.82
Average RNFL Thickness (μm)	96.2 ± 6.8	82.7 ± 7.3 ^a^	68.3 ± 9.1 ᵃᵇ	<0.001	0.79
Cup-to-Disk Ratio	0.35 ± 0.10	0.65 ± 0.12 ^a^	0.78 ± 0.09 ᵃᵇ	<0.001	0.78

Values are presented as mean ± standard deviation unless otherwise indicated. POAG = primary open-angle glaucoma; BCVA = best-corrected visual acuity; IOP = intraocular pressure; RNFL = retinal nerve fiber layer. * For gender, Cramer’s V is reported instead of η^2^. ᵃ Significantly different from control group (*p* < 0.05). ᵇ Significantly different from preperimetric POAG group (*p* < 0.05). Effect size interpretation: small (η^2^ = 0.01–0.05), medium (η^2^ = 0.06–0.13), large (η^2^ > 0.14).

## Data Availability

The dataset is available on request from the author.
